# Developing Culturally Appropriate Content for a Child-Rearing App to Support Young Children’s Socioemotional and Cognitive Development in Afghanistan: Co-Design Study

**DOI:** 10.2196/44267

**Published:** 2023-08-23

**Authors:** Haley M LaMonica, Jacob J Crouse, Yun J C Song, Mafruha Alam, Chloe E Wilson, Gabrielle Hindmarsh, Adam Yoon, Kelsie A Boulton, Mahalakshmi Ekambareshwar, Victoria Loblay, Jakelin Troy, Mujahid Torwali, Adam J Guastella, Richard B Banati, Ian B Hickie

**Affiliations:** 1 Youth Mental Health and Technology Team Brain and Mind Centre The University of Sydney Sydney Australia; 2 Clinic for Autism and Neurodevelopment Research Brain and Mind Centre The University of Sydney Sydney Australia; 3 The Australian Prevention Partnership Centre Sydney Australia; 4 Faculty of Arts and Social Sciences The University of Sydney Sydney Australia; 5 Medical Imaging Sciences Faculty of Medicine and Health Brain and Mind Centre, The University of Sydney Sydney Australia; 6 Australian Nuclear Science and Technology Organisation Sydney Australia

**Keywords:** child development, digital technology, global health, co-design, participatory research, stakeholder participation, mobile app, smartphone, mobile phone, Afghanistan

## Abstract

**Background:**

Optimal child-rearing practices can help mitigate the consequences of detrimental social determinants of health in early childhood. Given the ubiquity of personal digital technologies worldwide, the direct delivery of evidence-based information about early childhood development holds great promise. However, to make the content of these novel systems effective, it is crucial to incorporate place-based cultural beliefs, traditions, circumstances, and value systems of end users.

**Objective:**

This paper describes the iterative approach used to develop the Thrive by Five child-rearing app in collaboration with Afghan parents, caregivers (eg, grandparents, aunts, and nannies), and subject matter experts (SMEs). We outline how co-design methodologies informed the development and cultural contextualization of content to meet the specific needs of Afghan parents and the content was tested and refined in collaboration with key Afghan stakeholders.

**Methods:**

The preliminary content was developed based on a comprehensive literature review of the historical and sociocultural contexts in Afghanistan, including factors that influence child-rearing practices and early childhood development. After an initial review and refinement based on feedback from SMEs, this content was populated into a beta app for testing. Overall, 8 co-design workshops were conducted in July and August 2021 and February 2022 with 39 Afghan parents and caregivers and 6 SMEs to collect their feedback on the app and its content. The workshops were audio recorded and transcribed; detailed field notes were taken by 2 scribes. A theoretical thematic analysis using semantic codes was conducted to inform the refinement of existing content and development of new content to fulfill the needs identified by participants.

**Results:**

The following 4 primary themes were identified: child-rearing in the Afghan sociocultural context, safety concerns, emotion and behavior management, and physical health and nutrition. Overall, participants agreed that the app had the potential to deliver valuable information to Afghan parents; however, owing to the volatility in the country, participants recommended including more activities that could be safely done indoors, as mothers and children are required to spend most of their time at home. Additionally, restrictions on public engagement in music required the removal of activities referencing singing that might be performed outside the home. Further, activities to help parents reduce their children’s screen time, promote empathy, manage emotions, regulate behavior, and improve physical health and nutrition were requested.

**Conclusions:**

Direct engagement with Afghan parents, caregivers, and SMEs through co-design workshops enabled the development and refinement of evidence-based, localized, and contextually relevant child-rearing activities promoting healthy social, emotional, and cognitive development during the first 5 years of children’s lives. Importantly, the content was adapted for the ongoing conflict in Afghanistan with the aim of empowering Afghan parents and caregivers to support their children’s developmental potential despite the security concerns and situational stressors.

## Introduction

### The Importance of the First 5 Years of a Child’s Life

Globally, there is increased recognition of the importance of early childhood development for long-term health and well-being, which has led to greater investments in policies and programs. In 2015, the United Nations Sustainable Development Goals called for an emphasis on child well-being and the importance of nurturing care for children to reach their full developmental potential [1]. Further, the World Health Organization’s Nurturing Care Framework outlines the ability of countries, communities, and families to provide environments that ensure children’s access to health care and proper nutrition, protection from threats, and opportunities for early learning through responsive and emotionally supportive interactions with caregivers [2]. The Nurturing Care Framework recognizes the importance of universal access to evidence-based parenting education and support and promotes the delivery of parenting programs designed to support childhood health and well-being outcomes. A powerful context for nurturing care is the family home, wherein young children are most frequently cared for by not only mothers but also fathers and the extended family [3]. Importantly, cultural beliefs and behaviors are recognized as critical factors influencing child-rearing practices and the social context in which they occur [4].

### The Influence of Culture on Child-Rearing Practices

Although the definitions of culture vary, Kreuter and McClure [5] state that “social scientists generally agree that culture is learned, shared, transmitted intergenerationally, and reflected in a group’s values, beliefs, norms, practices, patterns of communication, familial roles, and other social regularities.” Further, it is readily accepted that culture influences child-rearing practices [6,7], including the beliefs and opinions about caregiving and child development, such as the skills and behaviors required of young children, expectations for when children should meet specific development milestones, and how and when to care for children [8]. This manifests in multiple ways, such as the application of the same parenting practice in different cultures for the same purpose (eg, child-directed speech to support language acquisition [9]) or contrasting interpretations of the function of a parenting behavior across different cultures (eg, harsh physical treatment can be used in initiation rites or viewed as abusive [8]). In low- and middle-income countries (LMICs), it is common for children to be raised by an extended network of family and community members, increasing the support for the primary caregiver and facilitating child supervision [10]. The extended family unit provides a degree of economic stability and a safety net to respond to life’s challenges as a group [11,12].

A child’s family is a microcosm of culture and is responsible for teaching, interpreting, and reinforcing cultural expectations during the early years of development. As such, parents’ beliefs, traditions, and value systems are embedded within their parenting practices, and child-rearing goals are also culturally driven [13]. Accordingly, parents adapt their parenting styles and behavioral responses to children in a culturally specific manner [14]. For instance (and recognizing that this is a generalization), literature on culture and family socialization practices indicates that parents from individualist communities are more likely to emphasize independence and autonomy, whereas parents from collectivist communities are more likely to value harmony and interdependence, each adapting their parenting practices to promote the development of these accepted values [15]. Mothers, in particular, are often seen as assuming roles within the family that are crucial for demonstrating cultural traditions, such as advisers, role models, protectors, and teachers of children [13]. Conversely, views on masculinity and the caregiving roles of fathers vary across cultures and are embedded within the dynamics of family relationships [16], resulting in varying levels of paternal investment and involvement [17]. Children learn culturally appropriate ways of emotional expression and regulation via socialization within the family and their broader social environment and through by-products of culture (eg, children’s books and approaches to schooling) [18].

Importantly, neither culture nor child-rearing practices are static. These concepts are impacted by a range of factors, including financial uncertainty, reduced gender gaps in education and employment, increased access to technology and the internet, and globalization [19]. Further, the interplay between culture and child-rearing may manifest differently across parents, family members of different generations, and geographic contexts [20]. Given the potential influence of family on the health and well-being of a young child, it is recommended that childhood development programs provide skill-building opportunities appropriate for multiple generations [3]. One way to do this is through the digitization of activities for parents and caregivers (eg, grandparents, aunts, and nannies), including via mobile health (mHealth) strategies.

### Delivering Educational Information to Parents and Caregivers via Digital Technologies

Most mHealth studies in LMICs have focused on improving maternal, newborn, and child survival rates, with a strong emphasis on nutritional outcomes and breastfeeding [21-23]. Only recently has there been an increased emphasis on mHealth strategies to support healthy early childhood development. Specifically, applications delivered to caregivers in both high-income countries and LMICs have promoted the development of motor [24], language [25], and cognitive abilities [26], as well as early childhood development more broadly [27].

Given the ubiquity of new digital technologies (particularly smartphones) worldwide, the mobile delivery of health and educational information holds great promise, including the potential to promote equity of access [28]. A review of 245 mHealth studies on reproductive, maternal, newborn, and child health from 2011 to 2016 found that 56.3% and 40% of the interventions were delivered via mobile apps or tablet-based applications and SMS text messages, respectively [29]. For mHealth interventions to be effective in LMICs, especially in rural or remote areas, they must have a supportive policy environment, a private-public partnership approach to developing and delivering the digital innovation, and a perception among end users and health care workers or support personnel facilitating the uptake of the technology that the technology is easy to use [30]. Conversely, barriers to the adoption of mHealth technologies and their content include the workload issues involved in the technology introduction and training for users, lack of technological infrastructure on a national scale, and absence of alternative content delivery methods (eg, hard copies of content and shared devices in publicly accessible facilities) [30].

### Study Context: Co-Designing the Thrive by Five International Program for Afghanistan

#### Thrive by Five International Program

Minderoo Foundation’s Thrive by Five International Program aims to empower parents with the knowledge they need to support the healthy development of their children from birth to 5 years of age and ensure universal access to this valuable parenting information regardless of socioeconomic status, literacy, gender, or other barriers (eg, digital literacy). As described in detail in the study by Crouse et al [31], the Youth Mental Health and Technology Team from the University of Sydney’s Brain and Mind Centre leads the development of the content for Thrive by Five. Importantly, the content is underpinned by a scientific framework developed by the research team that highlights 5 neurobiological systems fundamental to the development of behaviors and processes associated with social, emotional, and cognitive development, namely the stress response system, oxytocin system, learning system, fear-arousal-memory system, and circadian system. For more information about the relationship among these systems and early childhood development, please refer to the study by Crouse et al [31]. The Thrive by Five content is designed to target these key systems behaviorally to support healthy early childhood development. Specifically, the content includes a library of “Collective Actions” comprising scientific information about the principles of healthy early childhood development as well as child-rearing activities for parents, the extended family, and trusted members of the community to engage in with the children to support their socioemotional and cognitive development. The Thrive by Five app is the flagship product of the Thrive by Five International Program; however, a broader, multichannel approach to content dissemination is used, including distributing content via television and radio broadcasts, WhatsApp (Meta Platforms, Inc), medical centers, and hospitals.

Importantly, the Thrive by Five International Program is co-designed and developed in partnership with local parents, caregivers, and subject matter experts (SMEs) in each of the countries where it is implemented. Integral to this process in Afghanistan was the collaborative relationship with a local partner, that is, the Bayat Foundation. The Bayat Foundation is Afghanistan’s largest private philanthropic organization and a member of The Bayat Group, Afghanistan’s largest private diversified service company. Although based in the United States, the Bayat Foundation in association with The Bayat Group was founded by Afghan entrepreneurs Ehsanollah Bayat and Fatema Bayat and has contributed to >750 projects focused on improving the quality of life of children, women, people with low income, and older adults in Afghanistan. Specifically, the Bayat Foundation is dedicated to the health, education, and well-being of the people of Afghanistan, regardless of gender, age, ethnicity, marital status, or religion [32]. They seek to nourish the lives of Afghan citizens by providing for those in need and unlocking the potential of widows, women, children, youth, and men through programs and partnerships. Through such partnerships, they provide quality health care for women and newborns and seek to increase access to education through new or refurbished schools and scholarship programs to identify Afghanistan’s next generation of leaders. Additional objectives include economic empowerment through entrepreneurship and a collective focus on sustainable and ethical jobs, social justice, strengthened families, and cultural preservation. Importantly, as a key collaborative partner in this project, the Bayat Foundation was given access to all the content developed through this research to be able to disseminate it through their professional networks, which include both television and radio stations.

For mHealth initiatives, such as Thrive by Five, it is critical to understand the context, culture, behaviors, and attitudes of the people who are expected to use the digital health solutions [33]. The research team, including those with expert knowledge about Afghanistan (JT and MT), conducted a comprehensive literature review to develop an understanding of the historical and sociocultural contexts in Afghanistan. This involved gaining knowledge about cultural factors relevant to the research and development (R&D) context, including information about common child-rearing practices; family dynamics and relationships (eg, the role and position of the child, parents, siblings, and grandparents in the family); and broader social, economic, religious, and political factors that may influence family functioning and, in turn, early childhood development in Afghanistan. It is important to highlight that Afghanistan is a highly multicultural country comprising local tribes and ethnic groups, including Pashtuns, Tajiks, Hazaras, Uzbeks, Turkmen, and Aimaqs [34]. The Pashtun-based Taliban espouses rural Pashtun nationalism and conservative understandings of Islam, which may not align with the beliefs, customs, and practices of other Afghan citizens [35].

#### Collectivist Approaches to Child-Rearing

In Islamic states such as Afghanistan, culture and citizenship are underpinned by collectivism. Specifically, “individuals function through kinship groups where each individual within the group complements the other, thereby strengthening the unity of the group” [36]. The sanctity of the family structure is crucial to Afghan society, and the patriarchal kinship structure is legitimized by Islam, reinforcing the economic and political dependence of women on men [36]. To that end, within Afghanistan, the notion of the individual as distinct from the community does not exist [37]. Rather, an individual is defined based on their association with their family [38]. Thus, individuals strictly adhere to social norms, moral codes, and the identified interests of the family and community for the fear of bringing about disgrace or shame. Extended family kinship networks form the primary social and economic support systems [11]. At times of crisis, this can mean that a single-family compound may accommodate a large extended family, as obligation to family (ie, in-group solidarity) is above all else.

#### The Position of the Child in the Family

Children are considered critically important to the long-term welfare and continuity of families by most Afghan people [39] and are vessels of great hope and aspiration for many parents. Qualitative research in families across rural and urban Afghanistan revealed that parents typically hold positive feelings about their child (eg, love and care) and report a range of affectionate interactions with children, ultimately caring deeply about their child’s future [40]. The cultural concept of *tarbia* provides an important window into the position of children in the family, extended family, and wider community, especially pertaining to relationships with outsiders. Under the cultural and moral construct of *tarbia* children are expected to give respect and deference to parents and other elder family members, use appropriate spoken and body languages, act in a socially acceptable way, be polite, exercise good manners, be clean and well presented, and carry out various duties and tasks at the behest of elders [12,41]. Culturally, having *good tarbia* is seen to be essential to a *good life* and effective and successful relationships with people outside the family. The position of children at the center of the family and community with respect to *tarbia* is made evident by the high rates of physical punishment from families for not working toward *good tarbia* and the fact that men from other families are allowed to physically punish children who are not their own [40].

#### Gender Roles in Parenting

The family unit is arguably the most important institution in contemporary Afghan society. The extended family network is a major structure of family life, cohabitation, and social and economic support and a vehicle for cultural transmission and child-rearing [42]. The major family-based responsibilities among women include day-to-day activities involved in motherhood, nurturing their families, and socializing their children, which appear to be top priorities, even among women who have professional careers [12,39]. By contrast, men in the family home are seen as disciplinarians, maintaining the family’s economic welfare, advising children, and providing for aging parents [11]. However, interview-based studies of families living across rural and urban Afghanistan (Kabul, Herat, Bamiyan, and Nangarhar) conducted before the return of the Taliban rule, highlighted important differences between families in the expected roles of mothers and fathers in raising children. Although some families state that these responsibilities should be equal, others suggest that mothers pragmatically play a larger role because they are at home with children while fathers are working but that fathers nevertheless play an important role [40].

Since the Taliban regained control of Afghanistan in August 2021, women are generally confined to their homes, as they are forbidden from traveling without being accompanied by a man, which is likely to reaffirm the roles of women as daughters, wives, and mothers. Further, women are now largely denied the opportunity to contribute financially to the household, which can be dire in the absence of support from a husband or other male relatives. The extreme financial crisis in the country is resulting in an increase in child labor, including labor migration, child or early marriage, and the sale of children for trafficking [43].

#### Approaches to Education

In Afghanistan, there are few opportunities for early childhood education, which can lead to low literacy levels [43]. Indeed, Afghanistan has some of the lowest education levels in the world and a high percentage of illiteracy. A recent report by the United Nations Children’s Fund estimated that Afghanistan will lose US $5.4 billion in earning potential as a result of failing to educate girls [44]. Under the Taliban rule, girls have been prohibited from attending school beyond 6 years of age, and many are kept out of school because the teachers are men [43]. Notably, illiterate parents are likely to have lower expectations and aspirations regarding education for themselves and their children [45]. A study led by Qayumi et al [45] showed that educated parents were more inclined to consider the importance of early childhood education, supporting the view that the education of parents plays a vital role, impacting a child’s development and education. The lack of knowledge and understanding of the value of early childhood education by parents may, therefore, affect a child’s cognitive, physical, social, and emotional development. Given the limitations with regard to access to formal early childhood education, particularly for young girls, as well as low levels of literacy among some parents, there is a critical need to better inform parents and other caregivers about the importance of early childhood development and equip them with practical activities that they can engage in with their children to help fill this gap in the educational system.

### Objective

The mHealth landscape in Afghanistan is sparse. There are no apps focused on the social, emotional, and cognitive development of children in their first 5 years of life. Furthermore, the use of co-design methodologies in the Afghan context is novel. This paper highlights how we uniquely considered both the scientific principles of early childhood development and the sociocultural context of the target end users (ie, parents and caregivers in Afghanistan). We describe the process through which the Thrive by Five child-rearing app, the first of its kind in Afghanistan, and its content were developed for and in collaboration with Afghan parents, caregivers, and SMEs. Specifically, we highlight how the phased and iterative approach (1) supported the co-design and cultural contextualization of the content to meet the specific needs of Afghan parents and (2) facilitated the testing and refinement of the content before widespread implementation. Finally, we provide examples of the content that was collaboratively designed with and tailored to the needs of Afghan parents.

## Methods

### Co-Designing and Testing the Thrive by Five Content

**Figure 1:**
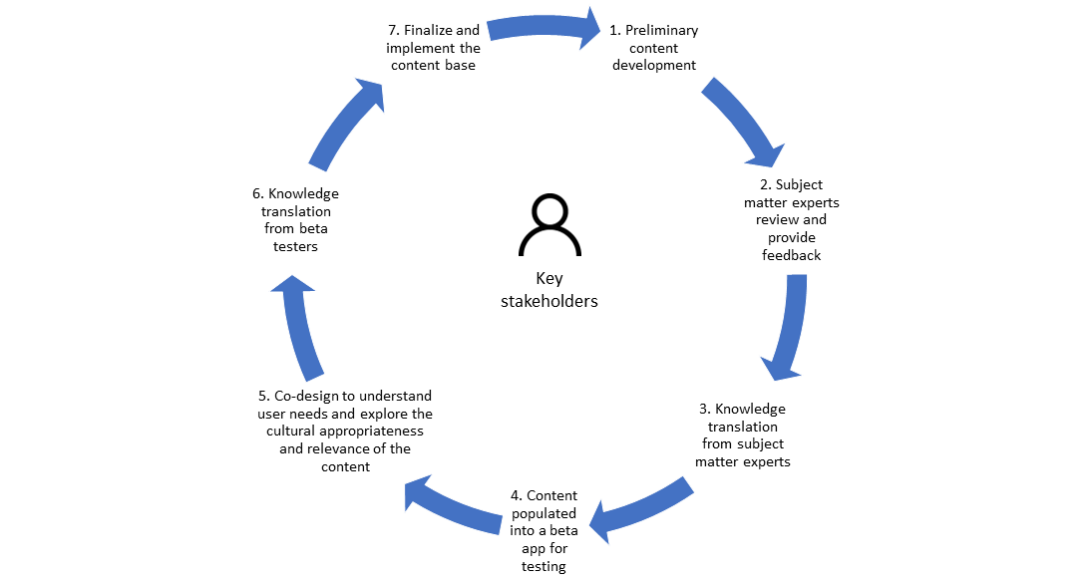
Research and development cycle (adapted from LaMonica et al [46], which is published under Creative Commons Attribution 4.0 International License [48]).

The iterative co-design process used in this study adhered to an established research protocol [46] adapted from the Medical Research Council’s Framework for Complex Interventions [47]. The R&D cycle, which includes the co-design process, is illustrated in Figure 1 and emphasizes the need for the iterative development and refinement of the content in collaboration with parents, caregivers, and SMEs. Importantly, this paper focuses solely on the co-design and development of the content for the Thrive by Five International Program and does not detail the development of the app features and functions.

**Figure 2:**
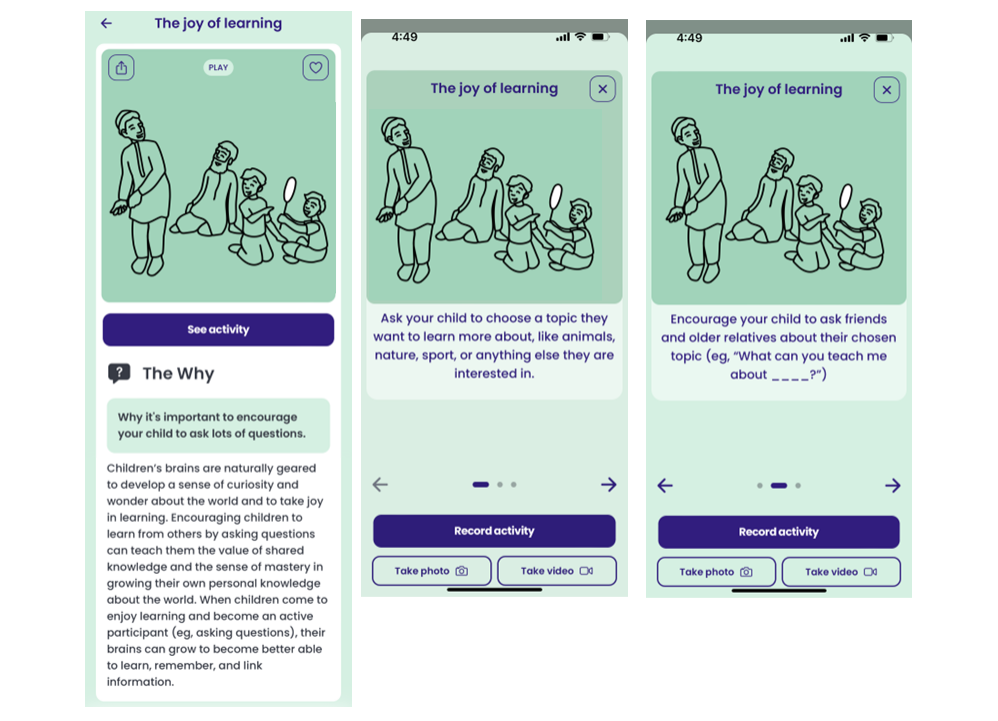
Example of a Collective Action: “The Why” and associated child-rearing activities.

Having developed an understanding of the cultures, traditions, and values of the Afghan people and the history and social context of the country, the research team developed a preliminary content base of 20 Collective Actions, which comprise 2 components: “The Why” and corresponding child-rearing activities. As highlighted in Figure 2, “The Why” reflects peer-reviewed scientific information related to child development, which is presented in an approachable, user-friendly language. Recognizing that raising a child takes place in a wider network of support beyond the immediate family, the child-rearing activities are for parents, the extended family, and trusted members of the community to engage in with the child to support their early development.

**Figure 3:**
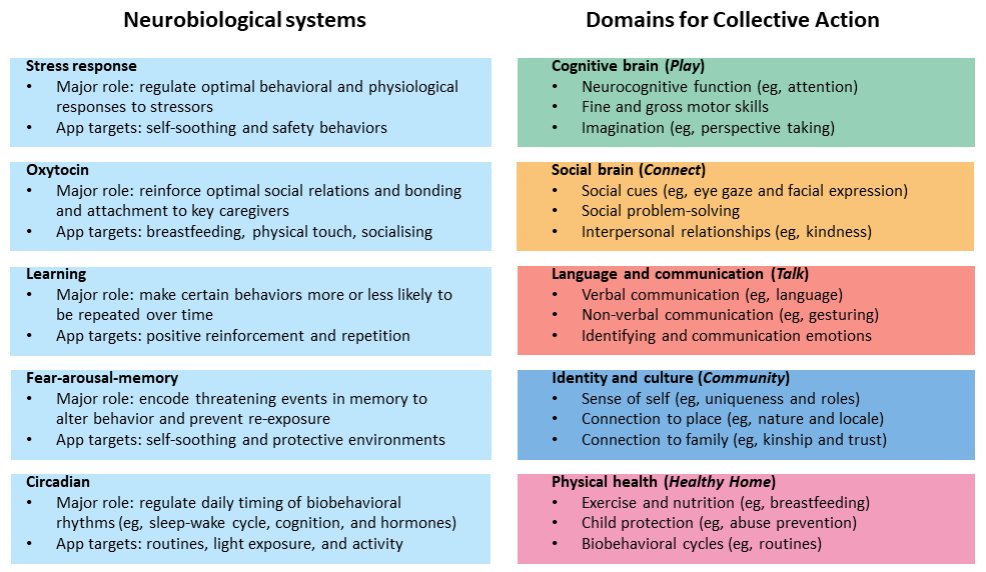
Neurobiological systems corresponding to the 5 domains of Collective Action (adapted from Crouse et al [31], which is published under Creative Commons Attribution 4.0 International License [48]).

As described in the study by Crouse et al [31] and presented in Figure 3, the content is organized into the following five domains relevant to social, emotional, and cognitive development based broadly on the Bright Tomorrows project (developed by Minderoo Foundation and Telethon Kids Institute): (1) “Play,” which refers to broad cognitive processes; (2) “Connect,” which relates to social skills and sociocognitive processes; (3) “Talk,” which refers to language and communication skills; (4) “Community,” which relates to the development of a sense of personal, social, and community identity; and (5) “Healthy home,” which refers to physical health, growth, and development. Importantly, the domain labels were designed to be approachable and user-friendly to users; however, Figure 3 highlights how these domains for Collective Action map to neurobiological systems fundamental to early childhood development. For further explanation of the scientific framework underpinning the content domains, please refer to the study by Crouse et al [31].

After the initial 20 Collective Actions were reviewed and refined in collaboration with the SME group (ie, specialists in early childhood development and education, psychology, and medicine) convened by the Bayat Foundation as well as representatives from the Bayat Foundation, a beta app was created for testing by potential real-world users, with all the content translated to Dari and Pashtu for the Afghan context and the review and validation of these translations was done by the team from the Bayat Foundation. Beta testers were invited to (1) test the content by using the app naturalistically (ie, in a manner of their own choosing) for a minimum of 1 week and (2) participate in a co-design workshop to provide their feedback on the relevance and cultural appropriateness of the content to enable iterative refinement.

### Qualitative Data Collection

As outlined in the study protocol by LaMonica et al [46], co-design workshops are central to the R&D cycle, allowing target end users the opportunity to provide feedback on the relevance and cultural appropriateness of the content. For inclusion in the workshops, participants had to be aged ≥18 years and have or care for at least 1 child (ie, classified as a parent or caregiver) or have subject matter expertise related to early childhood development (ie, classified as an SME). Participants were recruited through email and WhatsApp as well as via word of mouth by the site principal investigator from the Bayat Foundation using the organization’s professional networks.

Four 2-hour workshops with a total of 20 parents and caregivers (men: n=12, 60%; women: n=8, 40%) were conducted via Zoom (Zoom Video Communications, Inc) in July and August 2021; however, the co-design process was discontinued in August 2021 owing to the major conflict in the country. An additional 4 workshops were conducted in February 2022, specifically 3 with 19 parents and caregivers (men: n=10, 53%; women: n=9, 47%) and 1 with 6 SMEs (ie, medical doctors: n=2, 33%; dental hygienist: n=1, 17%; manager: n=1, 17%; teacher: n=1, 17%; clinical psychologist: n=1, 17%). The aim of the second round of workshops was to review and revalidate the cultural appropriateness and relevance of the content in the beta app before proceeding with the development of the full library of Collective Actions for Afghanistan. In particular, the researchers sought to understand how the content might need to be adapted to best suit parents and caregivers, considering the new social context and government policies. Considerations regarding the safety of parents and children were paramount in this process.

All workshops were cofacilitated by the University of Sydney researchers, Minderoo Foundation, BBE (ie, the Australian software development company), and *local champions* from the Bayat Foundation with strong connections with the community. Each workshop was divided into 2 parts. The first part was facilitated by BBE and focused on the app user experience, features, and functions; these data are not included in this paper. The second part, facilitated by the researchers using prompted discussion questions, explored how the content could be developed for and tailored to the needs of Afghan parents and caregivers. A sample agenda for the series of co-design workshops is available in Multimedia Appendix 1. Topics for discussion were sent to participants at least 1 week before the workshops to allow time for preparation for those who chose to do so.

The workshops supported the exploration of a number of critical areas, such as (1) the cultural relevance and appropriateness of the Collective Actions; (2) desired attributes, skills, and values for children; (3) information about children’s socioemotional and cognitive development that would be of benefit; and (4) essential people (family or other caregivers) in a child’s life who may benefit from the shared use of the app to support child-rearing. These research questions are critical to support the development of culturally appropriate content for the final product. In addition, the co-design work facilitated the discovery of areas for future development. Two scribes wrote detailed field notes in each workshop, including select verbatim transcription of dialogue of relevance to the co-design research. In addition, the workshops were recorded via Zoom and transcribed. A translator was present in the workshops to allow participants to communicate in either Dari or Pashtu. For a more detailed description of the full R&D cycle used in this project, including the co-design workshop methodology, please refer to the study protocol by LaMonica et al [46].

### Data Analysis, Outcomes, and Knowledge Translation

The interpretation of the qualitative data (ie, transcribed audio recordings and field notes) from the workshops followed a theoretical approach to thematic analysis using semantic codes aimed at informing content development and refinement [49]. All raw data across all participants were reviewed and checked by the research team to develop a coding framework outlining all the key concepts. Initial codes were derived from the research team’s comprehensive literature review of the sociocultural context in Afghanistan, including factors impacting early childhood development and child-rearing practices. Subsequently, these early codes were iteratively reviewed, discussed, revised, consolidated, and further specified by 3 researchers (MA, ME, and HML) with the support of workshop debriefing discussions with the broader co-design analysis team to develop the project codebook. The data were coded in NVivo (version 12; QSR International) software [50] using this framework by 2 researchers (MA and ME). As has been published previously, coding followed an established iterative process of reading, coding, and exploring the pattern and content of the coded data, followed by reflection and discussion to reach consensus [46].

Outcomes from the co-design process were examined by the research team to inform the revision of existing content such as the language and the Afghan examples used for the purposes of localization (eg, local songs, dances, games, and children’s stories). Further, new Collective Actions were developed to fill gaps in the content identified by parents, caregivers, and SMEs. Importantly, a fully revised library of >100 Collective Actions was developed before implementation, all of which were validated by the SME group and representatives of the Bayat Foundation.

### Ethics Approval

This study was approved by the University of Sydney’s Human Research Ethics Committee (protocol 2021/956). It is important to note that Afghanistan did not have a governing ethics body at the time of this study; therefore, approval from the University of Sydney’s Human Research Ethics Committee applied.

## Results

### Overview

Four primary themes were identified from the theoretical thematic analysis: (1) child-rearing in the Afghan sociocultural context, (2) safety concerns, (3) emotion and behavior management, and (4) physical health and nutrition. Overarching themes were derived from the comprehensive literature review of the Afghan sociocultural context and then further revised though the coding process. Single comments that represented an initial code or theme were also considered with regards to content development [51]. The analysis was conducted to inform the refinement of existing content and development of new content to fulfill the needs identified by participants. These themes are explored in greater detail in the subsequent subsections.

### Child-Rearing in the Afghan Sociocultural Context

The discussions with Afghan parents and SMEs highlighted the typical family structure, gender roles, routines, activities, and expectations of children, all of which aligned with our understanding of the study context. Specifically, Afghan families typically live in multigenerational households, in which children are frequently exposed to multiple languages (eg, Dari, Pashtu, and Arabic). Parents seek to instill good moral behavior in their children, including respect for elders and politeness, and an understanding of cultural norms and religious practices. Participants indicated that mothers are responsible for domestic tasks, including being the primary caregiver of children and older family members, with oversight and instruction often provided by mothers-in-law. Fathers generally contribute to educational and training activities conducted outside the home, such as those conducted at the local mosque. Many workshops focused on the time demands of mothers and their desire to have more support from fathers and other family members. It was noted that the daily routines of mothers can be quite stressful, as they are often responsible for caring for children (often multiple children) as well as extended family members in the household and managing domestic tasks and responsibilities. Encouragement of the involvement of fathers, siblings, and the extended family in activities was suggested by participants. For example, recognizing the benefits of reading to children, it was requested that the content encourage the involvement of elder family members in reading books or storytelling with children. Several Collective Actions were revised to explicitly encourage the involvement of fathers, siblings, and the extended family in activities (eg, reading and playing games).

Parents, caregivers, and SMEs requested the inclusion of religious content for children, such as the life story of Prophet Muhammed, verses from the Quran, Quranic lessons with correct pronunciation, and lessons on how to perform “salat” (prayer). As participation in religion can be an important way in which children learn about their culture and identity, the Collective Actions (eg, “Where do I belong?”) were updated to encourage the inclusion of children in religious and community festivals, such as Eid ul Fitar and Eid ul Adha, to build their social skills and help grow a sense of belonging to their community. However, specific religious teachings and materials were not included.

### Safety Concerns

Acknowledging the changing safety concerns within the country (since August 2021), several adaptations were suggested for the content, as outlined in Table 1. SMEs strongly urged that all content that referred to music, excluding Collective Actions involving singing in the home (eg, lullabies), be removed owing to restrictions on engaging in music in public. Further, it was noted that mothers and children are largely confined to their homes as a result of the instability and conflict in the country. As such, parents and SMEs suggested that the content include recommendations for indoor activities such as “Ludo King” or ball games that can be played within the safety of the home environment. The increased time spent indoors by children has translated into increased screen time. It was reported that mothers commonly used devices to occupy their children while they attended to domestic tasks. To combat this, parents and SMEs suggested that the content include more child-friendly indoor activities and games, encourage older siblings to read to and play with younger children, and suggest that grandparents and extended family members share stories with young children. Finally, parents sought strategies to manage their own stress and anxiety, as they acknowledged that this could have a negative impact on their parenting practices.

**Table 1:** Adapting the Thrive by Five content owing to safety concerns in Afghanistan in 2022.

Workshop observations	Changes to the content
“Parents need to be aware...some parents have phobia about letting children go outside and explore...There could be explosives and bombs outside...Parents need to warn children about picking up objects from the ground by themselves...unless an older adult is with them...this is from my own self-lived experiences when a ground item exploded...fortunately I didn’t pick it up...we need to consider the safety issue for children” (Expert, 2022). “...my son is 3 years old. when I go home, he asks me if he could go to the park or the local zoo, I cannot help him because the security is not that good” (Parent, 2022).	Activities that could only be performed outdoors were revised to include an indoor alternative (eg, climbing stairs or objects in the home instead of trees). Where possible, additional indoor activities were included.
“Singing (lullabies) is not a problem if we do it at home” (Parent, 2022).	To try to protect the safety of parents and children, all Collective Actions that referenced music (eg, “Moving to music,” “Let’s dance!” and “Feel the rhythm!”) that might be done outside the home were removed from the content library. Those that included singing in the home, however, were retained.
“Every day I tell my son that he shouldn’t play too many [online] games. My husband and I try to entertain him with something else like reading books or even television, he won’t listen. My kid wants to play hunting games, he is insistent on his ways and asks for the game” (Parent, 2022).	A new Collective Action, “Screen time,” which focuses on the potential impact of screen time on a child’s brain (eg, affecting sleep and circadian rhythms at night) and provides activities to decrease screen time, was introduced. In addition, multiple Collective Actions now include activities encouraging alternative indoor pastimes, such as reading books; games; and storytelling involving older siblings, fathers, and extended family (rather than using screens).
The current sociopolitical context in Afghanistan is uncertain and stressful for parents and other caregivers, including daily concerns regarding safety and security.	A new Collective Action, “Support for parents” was developed to help parents support each other and talk to those around them if they need support.

### Emotion and Behavior Management

Children’s behavior was another central point of discussion, particularly in the aftermath of the Taliban takeover. As highlighted in Table 2, parents sought ideas on how best to listen and respond to the emotional needs of their children with empathy and patience, create positive bonds, and manage stubbornness, including distress associated with having a new baby sibling. The Thrive by Five content was also regarded as a potential gateway for moral learning for young children. Parents and SMEs wanted content that would foster compassion for animals and a sense of responsibility for the environment among children. Finally, mothers, in particular, reflected on their upbringings and expressed a desire to approach parenting differently to promote confidence and social skills and instill values of compassion and empathy in their children.

**Table 2:** Developing the Thrive by Five content to support emotion and behavior management.

Workshop observations	Changes to the content
“Major problem is that most of the time children are irritated or upset and I don’t know how to calm them” (Parent, 2022).	One of the Collective Actions “Singing to baby” now encourages parents to manage their child’s emotions by singing calming, local songs, including “Aa Lalo Child Lalo” and “Allaha Sha Allaho,” to them.
Some parents noted problems with sibling rivalry and wanted advice about how to manage disruptive behavior from older children when a new baby is born.	As shown in Figure 4, a new Collective Action “Welcoming a new sibling” provides strategies for helping older children adjust to the arrival of a new baby sibling (eg, by helping parents wash the baby).
Cruelty toward animals was noted as a concern by parents, and they sought information as to how to raise compassionate children.	A new Collective Action “Being kind to animals” describes how children may be aggressive toward animals because they have seen others do this or they are yet to learn how to manage feelings such as anger. The activity suggests talking to children about how animals feel fear, anger, and distress, drawing pictures of animals or talking about animals from television shows (eg, Daniel’s Tiger Neighborhood), and intervening if one witnesses their child hurting an animal (and explaining that this behavior is not okay). The Collective Action “Caring for animals” was revised to include an activity entailing singing a song about the rights and safety of animals (“The Little Bird”).
Parents sought tips for positive parenting practices to instill confidence in their children. “I am trying not to be like my parents...I’ve always been punished for talking, I don’t want my child to be scared of anything” (Parent, 2021).	A new Collective Action “The joy of learning,” shown in Figure 2, was developed to encourage children to speak to other people (including older relatives) about a topic of interest to try to learn from them and gain confidence. In addition, “Standing up for myself and others” was developed to encourage children to stand up for themselves if they are feeling bullied.

**Figure 4:**
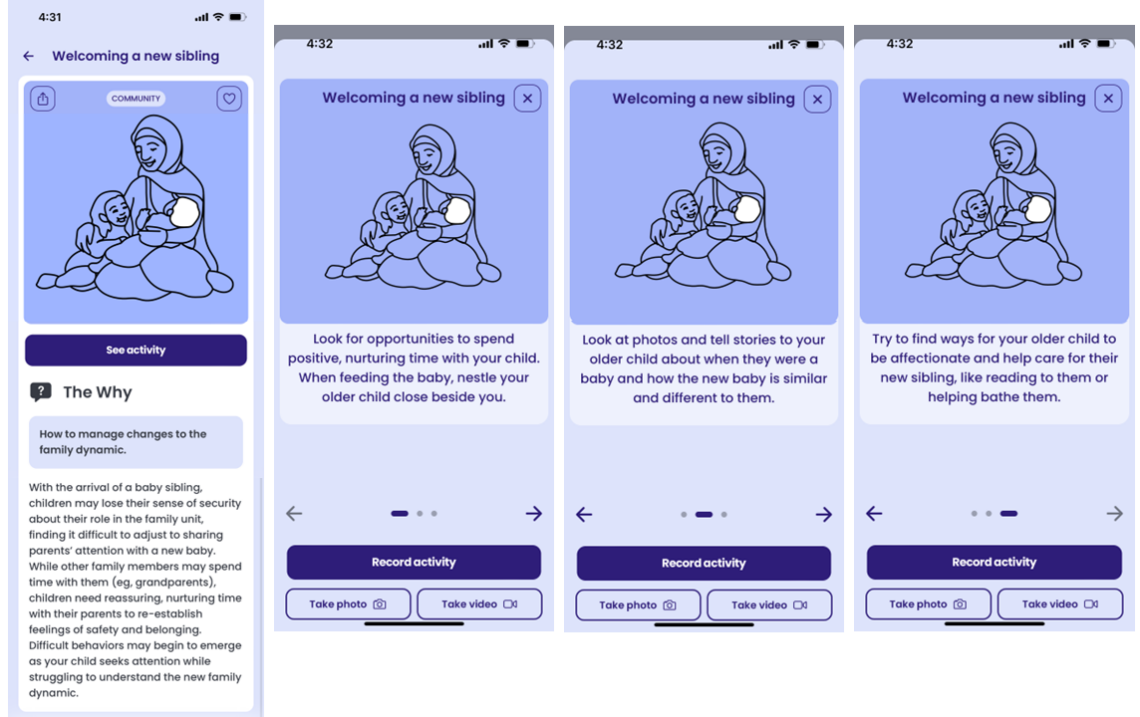
Example of a Collective Action co-designed with Afghan parents and caregivers.

### Physical Health and Nutrition

As shown in Table 3, parents, caregivers, and SMEs requested information to promote children’s physical health and nutrition. In relation to the latter, parents wanted to know the best complementary feeding practices, including what to feed infants when breastfeeding is inadequate or when children are weaning. Similarly, SMEs suggested that the content include a food chart to help parents and caregivers meet the nutritional requirements of their young children. Interestingly, there was a strong recommendation from SMEs that children be included in family meals to help them bond with family members and learn to eat independently. In addition, parents requested information about home remedies for common illnesses as well as contact information in case of a sudden pediatric emergency. Referencing the low vaccination coverage and absence of a formal vaccination campaign at present due to recent political changes, SMEs recommended the inclusion of vaccination schedules.

**Table 3:** Developing the Thrive by Five content to support physical health and development.

Workshop observations	Changes to the content
SMEs^a^ emphasized the need to better inform parents and caregivers about the nutritional needs of young children, such as through the provision of a food chart.	This information could not be accommodated into the content structure in the current app but was documented for consideration in future iterations.
Parents and caregivers noted that young children are typically excluded from family meals; however, SMEs suggested that mealtime was a valuable opportunity for children to bond with their extended family members.	A new Collective Action “Nutrition for children” was developed, which encourages enjoying meals as a whole family.
SMEs highlight the need to provide information about when children should be given different vaccinations. “Having a vaccination schedule or tracker is I think important for parents in Afghanistan. First, they do not know what vaccines should be taken at a certain time or age...since the purpose of this app is to improve parenting and child development” (Expert, 2022).	This information could not be accommodated into the content structure in the current app but was documented for consideration in future iterations.

^a^SME: subject matter expert.

## Discussion

### Principal Findings

This paper presents a first-of-its-kind study that aimed to co-design, test, and refine culturally appropriate and contextually relevant content for a child-rearing app to help Afghan parents and caregivers support the social, emotional, and cognitive development of children aged ≤5 years. Importantly, culturally informed research practices were used to guide the development of culturally relevant child-rearing content for the Afghan context [52].

On the basis of a recent systematic review, Jagtap [53] identified 10 guidelines fundamental to the design and development of solutions for marginalized or disadvantaged societies. First among them is the need to develop a holistic understanding of the sociocultural, economic, physical, and structural contexts of the target end user. To accomplish this, the literature regarding the study context was thoroughly reviewed by the research team, including 2 members of the research team (JT and MT) with specialized knowledge of the Afghan context. Creating this understanding of cultural factors, such as values, knowledge systems, and beliefs, was crucial for developing the preliminary content for the target audience. However, to avoid generalization based on the academic literature and account for the dramatic shifts in the sociopolitical landscape in Afghanistan beginning in 2021, it was vital that this project included the voices of Afghan parents, caregivers, and SMEs through participatory activities.

Co-design methodologies are a critical tool for including key stakeholders as active contributors in the design and development of digital solutions [54]. Through this collaboration, researchers develop an understanding of the sociocultural context, including the needs of and problems faced by the target audience [55]. Simultaneously, the involvement of target end users through co-design helps them develop a sense of ownership over the project. During the co-design workshops for this project, parents, caregivers, and SMEs agreed that the Thrive by Five app and its content had the potential to fill a knowledge gap in Afghanistan by providing scientific information about child-rearing activities to support nurturing caregiving practices. With regard to specific content, parents, caregivers, and SMEs highlighted the need to encourage the involvement of multiple caregivers (eg, fathers, grandparents, and older siblings) in child-rearing activities more explicitly to reduce the burden placed on mothers, who are typically the primary caregiver. Strategies for reducing the screen time of young children were requested, as devices had become popular tools for mothers to occupy children while they attended to domestic tasks. Further, it was recommended that the content include information about how to foster children’s self-confidence; help children protect themselves from bullying; strengthen children’s connection with their community; and manage children’s emotions and misbehavior, including cruelty toward animals and rivalry among siblings. Only by engaging directly with the target end users were we able to understand and appropriately address these immediate needs.

This study was conducted before and after the Taliban returned to power in Afghanistan in August 2021. As such, when co-designing the content with Afghan parents, caregivers, and SMEs, particular attention was paid to specific content adaptations required owing to the conflict and safety concerns in the country. Participants advised that all child-rearing activities needed to be safe for indoor settings, as children were now spending more time within the home as a result of the conflict. Further, it was strongly recommended that any activities that referenced singing that might be done outside the home be removed owing to restrictions on public engagement in singing. Finally, in the absence of professional support services, new content was requested to encourage parents and caregivers to support each other’s mental health and well-being. The latter is particularly critical, as war-related violence in conjunction with stressors such as poverty, food insecurity, unemployment, and limited social support is associated with poor mental health among parents [56]. Heightened parental stress due to prolonged conflict has been shown to be associated with an increased risk of family violence, including violence against children, in Afghanistan [57] as well as reductions in positive parent-child interactions and increased use of harsh discipline among Syrian refugees in Lebanon [58]. Importantly, parenting training programs conducted in communities impacted by conflict have been shown to improve both parenting [59] and child mental health outcomes [60]. Furthermore, there also appears to be a bidirectional relationship between parental psychosocial well-being and positive parenting practices, suggesting that improvements in parenting knowledge and skills may translate to improvements in the mental health of parents [61]. Therefore, child-rearing programs, such as Thrive by Five, have the potential to promote nurturing and responsive child-rearing practices to support improved outcomes in early childhood development as well as parental well-being.

This study highlights the need to account for the sociocultural context in the design and development of digital health solutions, such as the Thrive by Five app and its content. Specifically, the participatory methodologies helped researchers actively develop an understanding of how the Afghan social, cultural, and political contexts influence the conceptualizations of and priorities for the health and well-being of children in their early years [33]. Child-rearing practices are significantly influenced by culture [7], and it has been shown that effective multicultural parenting programs need to include culturally relevant content [62]. Importantly, solutions that are designed for but not with marginalized groups often fail to be adopted or sustained [53,63]. Therefore, collaborative engagement with Afghan parents, caregivers, and SMEs was critical to inform the development of culturally appropriate and contextually relevant content for Afghan parents and caregivers. Despite the current volatility in Afghanistan and its direct impact on child-rearing practices, technology offers an opportunity to empower Afghan parents and caregivers with information about nurturing child-rearing practices to help their children reach their full developmental potential, enable parents to obtain evidence-based and culturally appropriate information within the safety of their home environment, and expose fathers and extended family members to vital child-rearing information to support healthy development in the early years.

### Limitations

The conflicts and sociopolitical instability in Afghanistan limited the recruitment of a representative sample of Afghan parents and caregivers, including extended family members (eg, grandparents) and those from rural areas, differing provinces, and varying educational and socioeconomic backgrounds. Therefore, the feedback generated from the workshops should be interpreted with caution, as participants’ opinions may not be generalizable to the broader Afghan population. Further, most participants from the workshops in 2022 were unable to download the Thrive by Five app because of connectivity disruptions; hence, this group of parents did not practically test the app and its content at home with their children before providing feedback. As a result, the agendas for these workshops were adjusted to focus on participants’ needs, particularly considering the change in government in August 2021. The research team also recognizes that this project would have benefited from immersive fieldwork to develop a holistic understanding of the context; however, this was not possible owing to safety concerns and the funding agreement.

### Conclusions

Investment in strategies for promoting healthy early childhood development from a nurturing care perspective is relatively new in LMICs [3]. In the Afghan context, citizens have been deprived of education during decades of conflict and war, and many of their traditional values have been compromised by extreme ideologies for the fear of retribution, such as that from the Taliban [45]. As a result of the Taliban regaining control of Afghanistan, women have lost their right to engage in high school and university education or be employed outside the home [64]. Such restrictions highlight the need to ensure that all parents have access to knowledge about and an understanding of modern theories of child development and child-rearing practices to attempt to limit the loss of developmental potential among Afghan children [45]. Having been co-designed with parents and caregivers in Afghanistan, the Thrive by Five content addresses this need by disseminating evidence-based information and localized child-rearing activities that inform parents about how to support their child’s social, emotional, and cognitive development during the first 5 years. As such, Thrive by Five and its content are well placed to be a vital tool of education and empowerment for Afghan parents.

## Appendix

Multimedia Appendix 1 Co-design workshop agenda.

## Abbreviations

LMICs: low- and middle-income countries
mHealth: mobile health
R&D: research and development
SME: subject matter expert
