# Supplementary material for: Developing Culturally Appropriate Content for a Child-Rearing App to Support Young Children’s Socioemotional and Cognitive Development in Afghanistan: Co-Design Study
Source: JMIR Form Res. 2023 Aug 23;7:e44267. doi: 10.2196/44267 (PMC10483291; doi:10.2196/44267)
Supplement: Multimedia Appendix 1 [file formative_v7i1e44267_app1.docx]

**Multimedia Appendix 1: Co-Design Workshop Agenda (Prompted Discussion)**

**Content specific questions:**

1. What do you think about the activities, information, pictures, and language in the app? Anything you liked or did not like?
2. (i) What information would parents and/or caregivers in [country] want from the app to help care for children (0-5 years)?

(ii)Follow up: Are there any specific challenges (regarding parenting and child development) that we might be able to address through the app?

1. When you think back to being a first time parent (depending on number of children), what questions did you have? What did you want to know about raising a child?
2. In your family, who will be using this information (and app) most often?
3. Would any group of parents find the content more useful than the other (e.g. urban vs rural, working mum vs stay-at-home mum, mums vs dads, single mums)?

**Child development specific questions:**

1. What values or morals do you want to instill in children?
2. How do children learn about their culture and traditions (e.g. costumes, festivals, stories, plays etc.)?
3. What skills do you want your child to learn by the age of 5?
4. (i) Could you tell us about how children under the age of 5 spend their time during the day and before going to bed (first ask about weekdays and then about weekends)?
5. Follow up (if not covered in the above question): Who looks after children during the day and before they go to bed?
6. (If not covered in the two questions above) What is the daily schedule of an [country] parent/caregiver on a weekday and on weekends?
7. How do young children socialize with other children in their community?
   1. Do children have favourite games or activities that they do together?
8. What is the role of television and mobile phones in children’s day-to-day activities?

**Parenting related questions:**

1. (i) How do you manage tantrums and emotions of young children?
2. Follow up- Is there any parenting strategy that you like or found useful?
3. Do you receive parenting information or help from any other sources?
4. Is there anything you want to know from us?

**Taliban government questions:**

1. Is there anything in the content that is no longer appropriate under the Taliban government?
2. Are there any activities that cannot be done because of security concerns?
3. With Taliban government in mind, is there any specific information you would want included in the app to help you care for your children?
4. Given the increased stress for caregivers due to security concerns, is there any specific support or content for parents that should be included in the content?

**Access issues**

1. What kind of technology or devices do you and your family use? How is your access to the internet?
2. Do you think any special training is required to use the app?
3. When do you think parents would make the best use of the app (morning/day/night, when they are with the kid/not without the kid, weekends)?
4. Are there any issues (electricity, lack of smart phone) that would prevent parents from using the app?
5. For people unable to access the app, how can we deliver the information to them?
